# Carriage of carbapenemase‐ and extended‐spectrum cephalosporinase‐producing *Escherichia coli* and *Klebsiella pneumoniae* in humans and livestock in rural Cambodia; gender and age differences and detection of *bla*_OXA‐48 _in humans

**DOI:** 10.1111/zph.12612

**Published:** 2019-07-02

**Authors:** Clara Atterby, Kristina Osbjer, Viktoria Tepper, Elisabeth Rajala, Jorge Hernandez, Sokerya Seng, Davun Holl, Jonas Bonnedahl, Stefan Börjesson, Ulf Magnusson, Josef D. Järhult

**Affiliations:** ^1^ Zoonosis Science Center, Department of Medical Sciences Uppsala University Uppsala Sweden; ^2^ Division of Reproduction, Department of Clinical Sciences Swedish University of Agricultural Sciences (SLU) Uppsala Sweden; ^3^ Food and Agriculture Organization of the United Nations Phnom Penh Cambodia; ^4^ Institute of Environmental Engineering ETH Zürich Switzerland; ^5^ Center for Ecology and Evolution in Microbial Model Systems Linnaeus University Kalmar Sweden; ^6^ Department of Infectious Diseases, Kalmar County Council, Department of Clinical and Experimental Medicine Linköping University Linköping Sweden; ^7^ Diagnostic Centrum, Clinic Microbiologic Laboratory Kalmar County Hospital Kalmar Sweden; ^8^ General Directorate of Animal Health and Production Ministry of Agriculture, Forestry and Fisheries Phnom Penh Cambodia; ^9^ Department of Animal Health and Antimicrobial strategies National Veterinary Institute (SVA) Uppsala Sweden; ^10^ Department of Clinical and Experimental Medicine Linköping University Sweden

**Keywords:** AmpC, Cambodia, carbapenemase, colistin, ESBL, risk factors, rural population, zoonoses

## Abstract

**Objectives:**

This study investigates the frequency and characteristics of carbapenemase‐producing *Escherichia coli/Klebsiella pneumoniae* (CPE/K) and extended‐spectrum cephalosporinase‐producing *E. coli/K. pneumoniae* (ESCE/K) in healthy humans and livestock in rural Cambodia. Additionally, household practices as risk factors for faecal carriage of ESCE/K are identified.

**Methods:**

Faecal samples were obtained from 307 humans and 285 livestock including large ruminants, pigs and poultry living in 100 households in rural Cambodia in 2011. Each household was interviewed, and multilevel logistic model determined associations between household practices/meat consumption and faecal carriage of ESCE/K. CPE and ESCE/K were detected and further screened for colistin resistance genes.

**Results:**

CPE/K isolates harbouring *bla*_OXA‐48 _were identified in two humans. The community carriage of ESCE/K was 20% in humans and 23% in livestock. The same ESBL genes: *bla*_CTX‐M‐15_, *bla*_CTX‐M‐14_, *bla*_CTX‐M‐27_, *bl*a_CTX‐M‐55_, *bla*_SHV‐2_, *bla*_SHV‐12_, *bla*_SHV‐28_; AmpC genes: *bla*_CMY‐2_, *bla*_CMY‐42,_ *bla*_DHA‐1_; and colistin resistance genes: *mcr‐1‐like* and *mcr‐3‐like* were detected in humans and livestock. ESCE/K was frequently detected in women, young children, pigs and poultry, which are groups in close contact. The practice of burning or burying meat waste and not collecting animal manure indoors and outdoors daily were identified as risk factors for faecal carriage of ESCE/K.

**Conclusions:**

Faecal carriage of *E. coli* and *K. pneumoniae* harbouring extended‐spectrum cephalosporinase genes are common in the Cambodian community, especially in women and young children. Exposure to animal manure and slaughter products are risk factors for intestinal colonization of ESCE/K in humans.

**Impacts**

- Multidrug‐resistant *Escherichia coli* and *Klebsiella pneumoniae* harbouring cephalosporinase genes are common in rural Cambodian communities, especially in women, small children, poultry and pigs.
- The presence of cephalosporinase, carbapenemase and colistin resistance genes in bacteria from humans and livestock in Cambodian communities is worrying as such genes reduce the effectiveness of critically important antibiotics.
- Contact with animal manure and animal slaughter products enhance the risk of faecal colonization of multidrug‐resistant *E. coli* and *K. pneumoniae* in humans.

## 1 BACKGROUND

*Escherichia coli* and *Klebsiella pneumoniae* can cause a variety of severe infections, which are increasingly difficult to treat due to acquired resistance to critically important antibiotics (WHO, 2012). Resistance to broad‐spectrum beta‐lactam antibiotics in *E. coli* and *K. pneumoniae* is commonly due to the production of enzymes, which are characterized as carbapenemases, extended‐spectrum beta‐lactamases (ESBLs) and plasmid‐borne AmpC beta‐lactamases (AmpCs), and the latter two may also be collectively referred to as extended‐spectrum cephalosporinases (ESCs; Padmini, Ajilda, Sivakumar, & Selvakumar, 2017). Genes encoding carbapenemases, ESBLs and pAmpCs are often located on mobile genetic elements, for example plasmids, in *E. coli* and *K. pneumoniae*, enabling dissemination of antibiotic resistance genes between bacteria (Padmini et al., 2017). The emergence of carbapenemase‐producing *E. coli/K. pneumoniae* (CPE/K) and extended‐spectrum cephalosporinase‐producing *E. coli/K. pneumoniae* (ESCE/K) in livestock populations, environment and the community shows that transmission and persistence of such bacteria occur also outside of clinical settings (Guenther, Ewers, & Wieler, 2011).

Community carriage of CPE/K has only been described in a few countries such as Lebanon (Beyrouthy et al., 2014) and Spain (Rios, Lopez, Rodriguez‐Avial, Culebras, & Picazo, 2017), whereas community carriage of ESCE/K is common worldwide (Woerther, Burdet, Chachaty, & Andremont, 2013), with Southeast Asia identified as an area with a particularly high carriage of ESC‐producing Enterobacteriaceae (Hawkey, 2008; Karanika, Karantanos, Arvanitis, Grigoras, & Mylonakis, 2016; Woerther et al., 2013). In China and Thailand, community carriage of ESCE varies between 30% and 58% (Li et al., 2011; Ni et al., 2016; Zhou et al., 2015) and 58% and 62%, respectively (Niumsup et al., 2018; Sasaki et al., 2010). Several studies have indicated that foreign travel from countries with low ESBL frequency to Southeast Asia is a major risk factor for acquiring ESBL‐producing Enterobacteriaceae (Karanika et al., 2016). Other risk factors are recent use of antibiotics, recent hospitalization (Luvsansharav et al., 2012), owning a pet (Meyer, Gastmeier, Kola, & Schwab, 2012), household contact with infected community patients (Valverde et al., 2008) and regular consumption of chicken meat (Hijazi, Fawzi, Ali, & Abd El Galil, 2016b). Further potential risk factors that could influence the carriage of ESCE/K and CPE/K are household practices, especially in rural areas, involving poor food hygiene and living conditions that entail close contacts between humans, livestock and outdoor environment.

To date, there are no published reports of CPE/K in humans or livestock in Cambodia or of community carriage of ESCE/K in Cambodia. However, ESCE/K isolates have been detected as causative pathogens in Cambodian patients (Caron et al., 2018; Emary et al., 2015; Moore et al., 2016; Rammaert et al., 2012; Vlieghe et al., 2015). Genetic characterization of ESBL‐producing *E. coli* isolates in bloodstream infections in Cambodia revealed that 96% were of CTX‐M‐type, mainly *bla*_CTX‐M‐15 _and *bla*_CTX‐M‐14_ (Vlieghe et al., 2015). Little is known about the situation in Cambodian livestock, but one study detected *E. coli* isolates harbouring *bla*_TEM‐1 _and *bla*_CMY‐2 _from faecal samples from five pigs in a Cambodian slaughterhouse (Trongjit, Angkittitrakul, & Chuanchuen, 2016). Interviews with pig farmers have revealed that antibiotic use was common in pig farms (Ström, Boqvist, et al., 2018). ESBL‐producing *Salmonella* has recently been isolated from retail meat in Phnom Penh, Cambodia, and most isolates were harbouring *bla*_CTX‐M‐55 _(Nadimpalli et al., 2018).

The objectives of this study were to determine the detection frequency of carbapenem‐ and third‐generation cephalosporin‐resistant *E. coli *and *K. pneumoniae* in humans and livestock and to analyse whether household practices and meat consumption are potential risk factors associated with community carriage in rural Cambodia. Furthermore, we wanted to characterize the specific carbapenemase/extended‐spectrum cephalosporinase gene‐variants found and screen for colistin resistance genes and antibiotic susceptibility in CPE/K and ESCE/K isolates.

## 2 MATERIALS AND METHODS

### 2.1 Sampling

Samples were collected in Kampong Cham, Cambodia, in May 2011 from 10 households each in 10 villages as previously described (Osbjer et al., 2015). The sampling for this study was conducted in conjunction with other studies (Osbjer et al., 2017, 2015; Osbjer, Boqvist, et al., 2016; Osbjer, Tano, et al., 2016). Samples were collected from humans and 1–6 livestock from the same household. In total, 307 human samples from adult females (135), adult males (54), children 0–5 years (33) and children 6–15 years (85), and 280 livestock samples from cattle (80), water buffalo (23), pigs (39), ducks (28) and chicken (110). For statistical analysis, cattle and water buffalo were grouped as ruminants and chicken and ducks were grouped as poultry. 200 of the 308 human samples had been previously thawed twice before inclusion in this project.

### 2.2 Interviews

On day 1, the female head of the household was interviewed using a questionnaire, as previously described (Osbjer et al., 2015). Questions focused on eight household practices: (a) livestock home slaughter, (b) livestock access to sleeping and food preparation areas, (c) consumption of unsafe water (untreated well or pond water), (d) hand wash with soap after handling animals, (e) bury or burn meat waste products, (f) daily collection of animal manure indoors and outdoors, (g) hand wash with soap before and after cooking and (h) consumption of undercooked meat and meat consumption: the number of days each month that the household consumed pork, beef, fish, poultry and wild animals. Antibiotic use was not investigated in this study.

### 2.3 Isolation and characterization of CPE/K and ESCE/K

*Escherichia coli* and *K. pneumoniae* were isolated using three agar plates: chromID OXA‐48 (BioMérieux), chromID CARBA (BioMérieux) and CHROMagar C3G^R^ (Chromagar) and species identities were confirmed by matrix‐assisted laser desorption/ionization (MALDI) with time‐of‐flight mass spectrometry (TOF) according to previously described methods (Atterby et al., 2016). Identified isolates from the chromID OXA‐48 and chromID CARBA were subjected to multiplex‐PCR detecting carbapenemase gene‐groups *bla*_KPC_, *bla*_NDM_, *bla*_OXA_, *bla*_VIM_, *bla*_IMP_, *bla*_AIM_, *bla*_GIM_, *bla*_SIM_ and *bla*_DIM _with the specific variants determined by sequencing (Brink et al., 2013; Poirel, Walsh, Cuvillier, & Nordmann, 2011). Isolates from the CHROMagar C3G^R ^plates were subjected to multiplex‐PCRs detecting ESBL and pAmpC gene‐groups *bla*_CTX‐M_, *bla*_SHV_, *bla*_TEM_, *bla*_OXA‐1,_ *bla*_MOX_, *bla*_LAT_, *bla*_DHA_, *bla*_ACC_, *bla*_ACT _and *bla*_FOX‐1_, and the specific variants were determined by sequencing (Egervarn et al., 2014). All confirmed CPE/K and ESCE/K isolates were subjected to PCR targeting colistin resistance genes *mcr*‐1 to *mcr*‐5 (Rebelo et al., 2018). Isolates, in which no ESBL or AmpC genes could be identified, were further phenotypically tested according to EUCAST disc diffusion method for antimicrobial susceptibility testing (EUCAST, 2009) and double disc synergy test (Jarlier, Nicolas, Fournier, & Philippon, 1988). Isolates with AmpC phenotype were excluded due to suspected chromosomal AmpC‐production. Isolates with verified ESBL‐phenotype were characterized as ESCE/K and included in the data analysis and statistical tests. All CPE/K and ESCE/K isolates were tested for susceptibility to Ciprofloxacin, Trimethoprim‐sulfamethoxazole, Piperacillin/Tazobactam, Gentamicin, Meropenem, Tetracycline and Chloramphenicol using the EUCAST disc diffusion method and epidemiological cut‐offs (ECOFFs) (EUCAST, 2009), with the exception of tetracycline where the cut‐off was defined according to the normalized resistance interpretation method (Kronvall, Kahlmeter, Myhre, & Galas, 2003).

### 2.4 Data management and statistical analysis

#### 2.4.1 Detection frequency

Pearson's chi‐square tests were performed using GraphPad Prism version 8 to analyse differences in detection frequencies of ESCE/K and CPE/K between hosts. To control for multiple chi‐square tests on the livestock data (Ruminants vs. Pigs, Pigs vs. Poultry and Ruminants vs. Poultry), a Bonferroni correction (*p* ≤ 0.02) was used. In the human data (Adult females vs. Adult males and Children 0–5 years vs. Children 6–15 years), multiple tests were not performed, and thus, *p*‐values ≤0.05 were considered significant.

#### 2.4.2 Risk factors

Statistical analysis was performed in SAS for Windows 9.3 (SAS Institute Inc.).

The eight potential risk factors (Table 1) were screened using univariable logistic regression and selected for multivariable logistic regression if *p* < 0.2. A multivariable logistic regression model was used to investigate the association between faecal carriage of ESCE/K and potential risk or protective factors at individual level. Manual backward elimination was used until all remaining variables showed a *p* ≤ 0.05. The model was investigated for interactions between all included variables in the final model. The statistical models had three levels of nested factors in the hierarchy, where each person sampled was clustered within households that were clustered within villages. All variables in the model were categorical except for the continuous variable meat consumption.

**Table 1:** Origin and characterization of all carbapenemase‐ and extended‐spectrum cephalosporin‐producing *Escherichia coli* and *Klebsiella pneumoniae* detected in livestock and humans living in 10 rural villages in Kampong Cham, Cambodia, in 2011. In total, 307 human samples from adult females (135), adult males (54), children 0–5 years (33) and children 6–15 years (85), and 285 livestock samples from cattle (80), water buffalo (23), pigs (39), ducks (28) and chicken (110)

No.	Village. Household	Species	Sex/Age	Bacteria	Beta‐lactamase/ESBL/pAmpC/ carbapenemase gene	Colistin res gene	Additional gene group, not sequenced	Non‐wild‐type antibiotic susceptibility
1	01.01	Chicken	<1 year	*E. coli*	*bla*_CTX‐M27_		*bla*_TEM_	Tmp/Smx, Tc
2	01.03	Chicken	<1 year	*E. coli*	*bla*_CTX‐M55_		*bla*_TEM_	Ci, Tmp/Smx, Gm, Tc, Cm
3	01.09	Chicken	<1 year	*E. coli*	*bla*_CTX‐M15_		*bla*_TEM_	Ci, Tmp/Smx, Gm, Tc, Cm
4	01.10	Chicken	>1 year	*E. coli*	*bla*_CTX‐M27_		*bla*_TEM_	Tmp/Smx, Tc, Cm
5	02.02	Chicken	<1 year	*E. coli*	*bla*_CMY‐2_		*bla*_TEM_	
6	02.06	Chicken	>1 year	*E. coli*	*bla*_CMY‐2_		*bla*_TEM_	Ci, Tmp/Smx, Tc
7	02.06	Chicken	>1 year	*E. coli*	*bla*_CMY‐2_		*bla*_TEM_	Ci, Tmp/Smx, Tc
8	02.07	Chicken	<1 year	*E. coli*	*bla*_CTX‐M55_			Ci, Tc, Cm
9	02.08	Chicken	>1 year	*E. coli*	*bla*_CTX‐M15_		*bla*_TEM_, *bla*_OXA_	Ci, Tmp/Smx, Tc
10	03.03	Chicken	>1 year	*E. coli*	*bla*_CTX‐M55_			Tmp/Smx, Tc, Cm
11	03.04	Chicken	>1 year	*E. coli*	*bla*_CMY‐2_	*mcr‐3 like*	*bla*_TEM_	Ci, Tmp/Smx, Gm, Tc, Cm
12	04.05	Chicken	<1 year	*E. coli*	*bla*_CTX‐M55_			Tmp/Smx, Gm, Tc
13	04.05	Chicken	<1 year	*E. coli*	*bla*_CTX‐M55_		*bla*_TEM_	Tmp/Smx, Tzp, Tc
14	04.06	Chicken	>1 year	*E. coli*	*bla*_CTX‐M27_		*bla*_TEM_	Ci, Tmp/Smx, Gm
15	04.08	Chicken	>1 year	*E. coli*	*bla*_CTX‐M55_		*bla*_TEM_	Ci, Tmp/Smx, Gm, Tc
16	04.08	Chicken	>1 year	*E. coli*	*bla*_CTX‐M14_			Ci, Tc
17	04.08	Chicken	>1 year	*E. coli*	*bla*_CMY‐2_		*bla*_TEM_	Ci, Tmp/Smx, Tc, Cm
18	04.10	Chicken	<1 year	*E. coli*	*bla*_CTX‐M14_	*mcr‐1 like*	*bla*_TEM_	Ci, Tmp/Smx, Gm, Tc
19	04.10	Chicken	>1 year	*E. coli*	*bla*_CTX‐M14_	*mcr‐1 like*	*bla*_TEM_	Tmp/Smx, Gm
20	05.02	Chicken	>1 year	*E. coli*	*bla*_CTX‐M15_, *bla*_CTX‐M27_		*bla*_TEM_	Tmp/Smx, Tc
21	05.02	Chicken	>1 year	*E. coli*	*bla*_CTX‐M14_			
22	06.03	Chicken	<1 year	*E. coli*	*bla*_CTX‐M55_			Ci, Tmp/Smx, Gm, Tc
23	08.05	Chicken	<1 year	*E. coli*	*bla*_CTX‐M15_		*bla*_OXA_	Ci, Tmp/Smx, Tzp, Tc, Cm
24	08.05	Chicken	<1 year	*E. coli*	*bla*_CTX‐M15_		*bla*_TEM_, *bla*_OXA_	Ci, Tmp/Smx, Tzp, Tc, Cm
25	08.06	Chicken	<1 year	*E. coli*	*bla*_CTX‐M55_			Ci, Tmp/Smx, Tc, Cm
26	08.07	Chicken	>1 year	*E. coli*	*bla*_CTX‐M14_		*bla*_TEM_	Ci, Tmp/Smx, Tc, Cm
27	08.10	Chicken	<1 year	*E. coli*	*bla*_CTX‐M55_, *bla*_CTX‐M27_		*bla*_TEM_	Ci, Tmp/Smx, Tc, Cm
28	08.10	Chicken	<1 year	*E. coli*	*bla*_CTX‐M55_		*bla*_TEM_	Ci, Tmp/Smx, Tc, Cm
29	08.10	Chicken	<1 year	*E. coli*	*bla*_CTX‐M27_		*bla*_TEM_	Mer
30	09.03	Chicken	>1 year	*E. coli*	*bla*_CTX‐M14_			Ci, Tmp/Smx, Tc
31	09.05	Chicken	>1 year	*E. coli*	*bla*_CTX‐M15_		*bla*_TEM_, *bla*_OXA_	Ci, Tmp/Smx, Tzp, Gm, Tc
32	09.09	Chicken	<1 year	*E. coli*	*bla*_CTX‐M27_		*bla*_TEM_	Tmp/Smx, Tc, Cm
33	10.01	Chicken	<1 year	*E. coli*	*bla*_CTX‐M27_, *bla*_CMY‐2_		*bla*_TEM_	Ci, Tmp/Smx, Tc, Cm
34	10.10	Chicken	>1 year	*E. coli*	*bla*_CMY‐2_			
35	01.10	Duck	>1 year	*E. coli*	*bla*_CTX‐M55_			Ci, Tmp/Smx, Tc, Cm
36	02.03	Duck	>1 year	*E. coli*	*bla*_CTX‐M14_	*mcr‐1 like*	*bla*_TEM_	Ci, Tmp/Smx, Gm, Tc
37	02.05	Duck	>1 year	*E. coli*	*bla*_CTX‐M55_		*bla*_TEM_	Tmp/Smx, Tc, Cm
38	04.09	Duck	>1 year	*E. coli*	*bla*_CMY‐2_		*bla*_TEM_	Ci, Tmp/Smx, Tc, Cm
39	01.10	Pig	>6 months	*E. coli*	*bla*_CTX‐M55_		*bla*_TEM_	Tmp/Smx, Tc
40	02.03	Pig	<3 month	*E. coli*	*bla*_CTX‐M27_			Ci, Tmp/Smx, Gm, Tc
41	02.09	Pig	<3 month	*E. coli*	*bla*_CMY‐2_	*mcr‐3 like*	*bla*_TEM_	Ci, Tmp/Smx, Tc, Cm
42	03.01	Pig	>6 months	*E. coli*	*bla*_CTX‐M55_			Tmp/Smx, Tc, Cm
43	03.02	Pig	>6 months	*E. coli*	*bla*_CTX‐M27_			Ci, Tmp/Smx, Tzp, Tc, Cm
44	03.03	Pig	>6 months	*E. coli*	*bla*_CTX‐M27_		*bla*_TEM_	Tmp/Smx, Tc, Cm
45	03.03	Pig	>6 months	*E. coli*	*bla*_CTX‐M27_		*bla*_TEM_	Ci, Tmp/Smx, Gm, Tc, Cm
46	03.07	Pig	3–6 month	*E. coli*	*bla*_CTX‐M14_	*mcr‐1 like*	*bla*_TEM_	Tmp/Smx, Gm, Tc, Cm
47	04.01	Pig	>6 months	*E. coli*	*bla*_CTX‐M55_			Tmp/Smx, Tc, Cm
48	04.03	Pig	3–6 month	*E. coli*	*bla*_CTX‐M55_			Tmp/Smx, Tc, Cm
49	04.06	Pig	3–6 month	*E. coli*	*bla*_CTX‐M27_			Ci, Tmp/Smx, Tc, Cm
50	04.06	Pig	3–6 month	*E. coli*	*bla*_CTX‐M55_		*bla*_TEM_	Tmp/Smx, Tc, Cm
51	04.06	Pig	3–6 month	*E. coli*	*bla*_CTX‐M15_		*bla*_OXA_	Ci, Tmp/Smx, Tzp, Gm, Tc
52	04.07	Pig	>6 months	*E. coli*	*bla*_CMY‐2_		*bla*_TEM_	Tmp/Smx, Tc, Cm
53	04.10	Pig	<3 month	*E. coli*	*bla*_CTX‐M14_	*mcr‐1 like*	*bla*_TEM_	Tmp/Smx, Tc, Cm
54	06.02	Pig	<3 month	*E. coli*	*bla*_CMY‐2_	*mcr‐1 like*	*bla*_TEM_	Ci, Tmp/Smx, Gm, Tc, Cm
55	06.09	Pig	<3 month	*E. coli*	*bla*_CTX‐M14_			Ci, Tmp/Smx, Gm, Tc, Cm
56	09.05	Pig	<3 month	*E. coli*	*bla*_CTX‐M27_		*bla*_TEM_	Ci, Tmp/Smx, Tzp, Tc, Cm
57	01.02	Cattle	>2 years	*E. coli*	*bla*_CTX‐M55_		*bla*_TEM_	Ci, Tmp/Smx, Gm, Tc, Cm
58	02.06	Cattle	<6 month	*E. coli*	*bla*_CMY‐2_		*bla*_TEM_	Ci, Tmp/Smx, Tc
59	09.04	Cattle	>2 years	*E. coli*	*bla*_CTX‐M15_		*bla*_TEM_	Ci, Tmp/Smx, Tc
60	09.04	Cattle	>2 years	*E. coli*	*bla*_CTX‐M15_		*bla*_TEM_, *bla*_OXA_	Ci, Tmp/Smx, Tzp, Gm, Tc
61	09.09	Cattle	>2 years	*E. coli*	*bla*_CTX‐M55_			Ci, Cm
62	03.06	Ruminant	<6 month	*E. coli*	*bla*_CTX‐M55_		*bla*_TEM_	Ci, Tmp/Smx, Tc, Cm
63	07.01	Buffalo	>2 years	*E. coli*	*bla*_CTX‐M27_		*bla*_TEM_	Ci, Tmp/Smx, Tc, Cm
64	01.01	Female	Adult	*E. coli*	*bla*_CTX‐M27_		*bla*_TEM_	Ci, Tmp/Smx, Te
65	02.03	Female	Adult	*E. coli*	*bla*_CTX‐M27_		*bla*_TEM_	Ci, Tmp/Smx, Te
66	02.10	Female	Adult	*E. coli*	*bla*_CTX‐M14_		*bla*_TEM_	Tmp/Smx, Te, Cm
67	03.01	Female	Adult	*E. coli*	*bla*_CTX‐M55_		*bla*_TEM_	Ci, Tmp/Smx, Te, Cm
68	03.06	Female	Adult	*E. coli*	*bla*_CTX‐M55_	*mcr‐3 like*	*bla*_TEM_	Tmp/Smx, Te, Cm
69	04.08	Female	Adult	*E. coli*	*bla*_CTX‐M27_		*bla*_TEM_	Ci, Tmp/Smx, Te, Cm
70	04.10	Female	Adult	*E. coli*	*bla*_CTX‐M15_		*bla*_OXA_	Ci, Tmp/Smx, Tzp, Gm, Te
71	05.01	Female	Adult	*E. coli*	*bla*_CTX‐M14_		*bla*_TEM_	Te, Cm
72	05.03	Female	Adult	*E. coli*	*bla*_CTX‐M27_		*bla*_TEM_	Tmp/Smx, Te
73	05.09	Female	Adult	*E. coli*	*bla*_CTX‐M27_		*bla*_TEM_	Tmp/Smx, Te, Cm
74	06.10	Female	Adult	*E. coli*	*bla*_CTX‐M27_			Tmp/Smx, Te
75	07.01	Female	Adult	*E. coli*	*bla*_CTX‐M14_		*bla*_TEM_	Ci, Tmp/Smx, Te, Cm
76	07.04	Female	Adult	*E. coli*	*bla*_CMY‐2_		*bla*_TEM_	Ci, Tmp/Smx, Te, Cm
77	07.04	Female	Adult	*E. coli*	*bla*_CTX‐M27_		*bla*_TEM_	Ci, Tmp/Smx, Te, Cm
78	07.05	Female	Adult	*E. coli*	*bla*_CTX‐M27_		*bla*_TEM_	Ci
79	07.08	Female	Adult	*E. coli*	*bla*_CMY‐2_, *bla*_OXA‐48_		*bla*_TEM_	Ci, Tmp/Smx, Tzp, Te, Mer
80	07.08	Female	Adult	*E. coli*	*bla*_CTX‐M55_		*bla*_TEM_	Tmp/Smx, Te, Cm
81	08.01	Female	Adult	*E. coli*	*bla*_CMY‐2_		*bla*_TEM_	Ci, Tmp/Smx, Tzp, Gm, Te, Cm
82	08.04	Female	Adult	*E. coli*	*bla*_CTX‐M14_, *bla*_OXA‐48_			Tmp/Smx, Tzp, Mer
83	08.09	Female	Adult	*E. coli*	*bla*_CTX‐M55_		*bla*_TEM_	Ci, Tmp/Smx, Gm, Te, Cm
84	09.02	Female	Adult	*E. coli*	*bla*_CMY‐42_			Ci, Tmp/Smx, Tzp, Te, Cm
85	09.03	Female	Adult	*E. coli*	*bla*_CTX‐M15_			Ci, Tmp/Smx, Cm
86	09.04	Female	Adult	*E. coli*	Unknown			Te, Cm
87	09.07	Female	Adult	*E. coli*	*bla*_CTX‐M27_		*bla*_TEM_	Ci, Tmp/Smx, Te, Cm
88	10.02	Female	Adult	*E. coli*	*bla*_CTX‐M55_		*bla*_TEM_	Ci, Tmp/Smx, Gm, Te, Cm
89	10.09	Female	Adult	*E. coli*	*bla*_CTX‐M27_		*bla*_TEM_	Tmp/Smx, Tzp, Te, Cm
90	03.07	Male	Adult	*E. coli*	*bla*_CTX‐M27_		*bla*_TEM_	Ci, Tmp/Smx, Gm, Te
91	04.05	Male	Adult	*E. coli*	*bla*_CTX‐M27_		*bla*_TEM_	Tmp/Smx, Gm, Te, Cm
92	06.03	Male	Adult	*E. coli*	*bla*_CTX‐M55_			Ci, Tmp/Smx, Gm, Te
93	06.04	Male	Adult	*E. coli*	*bla*_CTX‐M15_		*bla*_OXA_	Ci, Tmp/Smx, Tzp, Gm, Te
94	07.03	Male	Adult	*E. coli*	*bla*_CTX‐M15_		*bla*_OXA_	Ci, Tmp/Smx, Tzp, Gm, Te
95	07.05	Male	Adult	*E. coli*	*bla*_CTX‐M27_		*bla*_TEM_	
96	01.09	Child	6–15 years	*E. coli*	*bla*_CTX‐M14_		*bla*_OXA_	Ci, Tmp/Smx, Tzp, Te, Cm
97	03.06	Child	6–15 years	*E. coli*	*bla*_CTX‐M55_		*bla*_TEM_, *bla*_OXA_	Ci, Tmp/Smx, Tzp, Te, Cm
98	03.06	Child	6–15 years	*E. coli*	*bla*_CTX‐M55_		*bla*_TEM_	Tmp/Smx, Te, Cm
99	03.10	Child	6–15 years	*E. coli*	*bla*_CTX‐M14_	*mcr‐1 like*	*bla*_TEM_	Ci, Gm, Te, Cm
100	05.03	Child	6–15 years	*E. coli*	*bla*_CTX‐M27_		*bla*_TEM_	Tmp/Smx, Te
101	05.04	Child	6–15 years	*E. coli*	*bla*_CTX‐M55_			Gm, Te
102	07.01	Child	6–15 years	*E. coli*	*bla*_CTX‐M14_			Tmp/Smx, Te
103	07.03	Child	6–15 years	*E. coli*	*bla*_CTX‐M55_		*bla*_TEM_	Tmp/Smx, Te, Cm
104	07.06	Child	6–15 years	*E. coli*	*bla*_CTX‐M27_		*bla*_TEM_	Tmp/Smx, Te
105	07.08	Child	6–15 years	*E. coli*	*bla*_CTX‐M55_		*bla*_TEM_	Tmp/Smx, Te, Cm
106	08.08	Child	6–15 years	*E. coli*	*bla*_CTX‐M55_		*bla*_TEM_	Ci, Tmp/Smx, Gm, Te, Cm
107	03.03	Child	2–5 years	*E. coli*	*bla*_CTX‐M27_		*bla*_TEM_	Ci, Tmp/Smx, Te, Cm
108	04.06	Child	2–5 years	*E. coli*	*bla*_CTX‐M55_		*bla*_TEM_	Ci, Tmp/Smx, Te, Cm
109	04.06	Child	2–5 years	*E. coli*	*bla*_CTX‐M14_		*bla*_TEM_	Ci, Tmp/Smx, Te, Cm
110	05.07	Child	2–5 years	*E. coli*	*bla*_CTX‐M15_		*bla*_OXA_	Ci, Tmp/Smx, Tzp, Gm, Te
111	06.03	Child	2–5 years	*E. coli*	*bla*_CTX‐M14_		*bla*_TEM_	Te, Cm
112	07.01	Child	2–5 years	*E. coli*	*bla*_CTX‐M15_			Ci, Tmp/Smx, Tzp, Gm, Te
113	06.10	Child	<2 years	*E. coli*	*bla*_CTX‐M14_			Ci, Tmp/Smx, Gm, Te, Cm
114	08.01	Child	<2 years	*E. coli*	*bla*_CTX‐M55_, *bla*_CTX‐M14_, *bla*_CMY‐2_		*bla*_TEM_	Ci, Tmp/Smx, Tzp, Gm, Te, Cm
115	10.03	Child	<2 years	*E. coli*	*bla*_CTX‐M55_		*bla*_TEM_	Ci, Tmp/Smx, Tzp, Te
116	03.06	Human	Unknown	*E. coli*	*bla*_CTX‐M55_		*bla*_TEM_	Tmp/Smx, Te, Cm
117	02.03	Chicken	>1 year	*Kl. pn.*	*bla*_SHV‐12_			Ci, Tmp/Smx, Tc, Cm
118	03.03	Chicken	>1 year	*Kl. pn.*	*bla*_CTX‐M14_	*mcr‐3 like*	*bla*_SHV_	Ci, Tmp/Smx, Gm, Tc, Cm
119	05.02	Chicken	<1 year	*Kl. pn.*	*bla*_SHV‐2_			Ci, Tmp/Smx, Gm, Tc, Cm
120	05.07	Chicken	<1 year	*Kl. pn.*	*bla*_SHV‐1_			Tmp/Smx, Tc, Cm
121	09.07	Duck	<1 year	*Kl. pn.*	*bla*_DHA‐1_		*bla*_SHV_, *bla*_OXA_	Ci, Tmp/Smx, Tzp
122	03.06	Female	Adult	*Kl. pn.*	*bla*_SHV‐28_			Tmp/Smx, Tzp, Te, Cm
123	04.07	Female	Adult	*Kl. pn.*	*bla*_SHV‐2_			Ci, Tmp/Smx, Gm, Te, Cm
124	04.08	Female	Adult	*Kl. pn.*	*bla*_SHV‐2_			Ci, Tmp/Smx, Tzp, Gm, Te, Cm
125	07.04	Female	Adult	*Kl. pn.*	*bla*_CTX‐M15_		*bla*_SHV_, *bla*_TEM_, *bla*_OXA_	Ci, Tmp/Smx, Tzp, Gm, Te
126	07.08	Female	Adult	*Kl. pn.*	*bla*_SHV‐2_		*bla*_TEM_	Ci, Tmp/Smx, Gm, Te, Cm
127	08.04	Female	Adult	*Kl. pn.*	*bla*_DHA‐1_, *bla*_OXA‐48_, *bla*_SHV‐11_			Ci, Tmp/Smx, Tzp, Cm, Mer
128	08.04	Female	Adult	*Kl. pn.*	*bla*_CTX‐M14_		*bla*_SHV_	Ci, Tmp/Smx, Te
129	09.02	Female	Adult	*Kl. pn.*	*bla*_SHV‐1_			Tmp/Smx, Te, Cm
130	10.01	Female	Adult	*Kl. pn.*	*bla*_DHA‐1_, *bla*_SHV‐2_		*bla*_TEM_	Tmp/Smx
131	07.08	Child	6–15 years	*Kl. pn.*	*bla*_CTX‐M27_		*bla*_SHV_	Ci, Tmp/Smx, Te, Cm
132	06.04	Child	2–5 years	*Kl. pn.*	*bla*_SHV‐1_			Tmp/Smx, Gm, Te, Cm

Abbreviations: Ci, ciprofloxacin; Cm, chloramphenicol; Gm, Gentamicin; *Kl. pn., Klebsiella pneumoniae*; Mer, Meropenem; Te, Tetracycline; Tmp/Smx, sulfamethoxazole/trimethoprim; Tzp, piperacillin/tazobactam.

### 2.5 Study approval

Ethical approval (43 NECHR, 8th April 2011) was obtained prior to the survey from the National Ethics Committee for Health Research, Ministry of Health, Cambodia, and an advisory ethical statement (Dnr 2011/63) was obtained from the Regional Board for Research Ethics in Uppsala, Sweden. The authors assert that all procedures contributing to this work comply with the ethical standards of the relevant national and institutional committees on human experimentation and with the Helsinki Declaration of 1975, as revised in 2008.

## 3 RESULTS

Faecal samples from 307 humans and 285 livestock living in 100 households in 10 villages in Kampong Cham province, Cambodia, were collected and analysed for the presence of carbapenem‐ and third‐generation cephalosporin‐resistant *E. coli *and *K. pneumoniae*.

### 3.1 Determination of carbapenemase, extended‐spectrum cephalosporinase and colistin resistance genes

**Figure 1:**
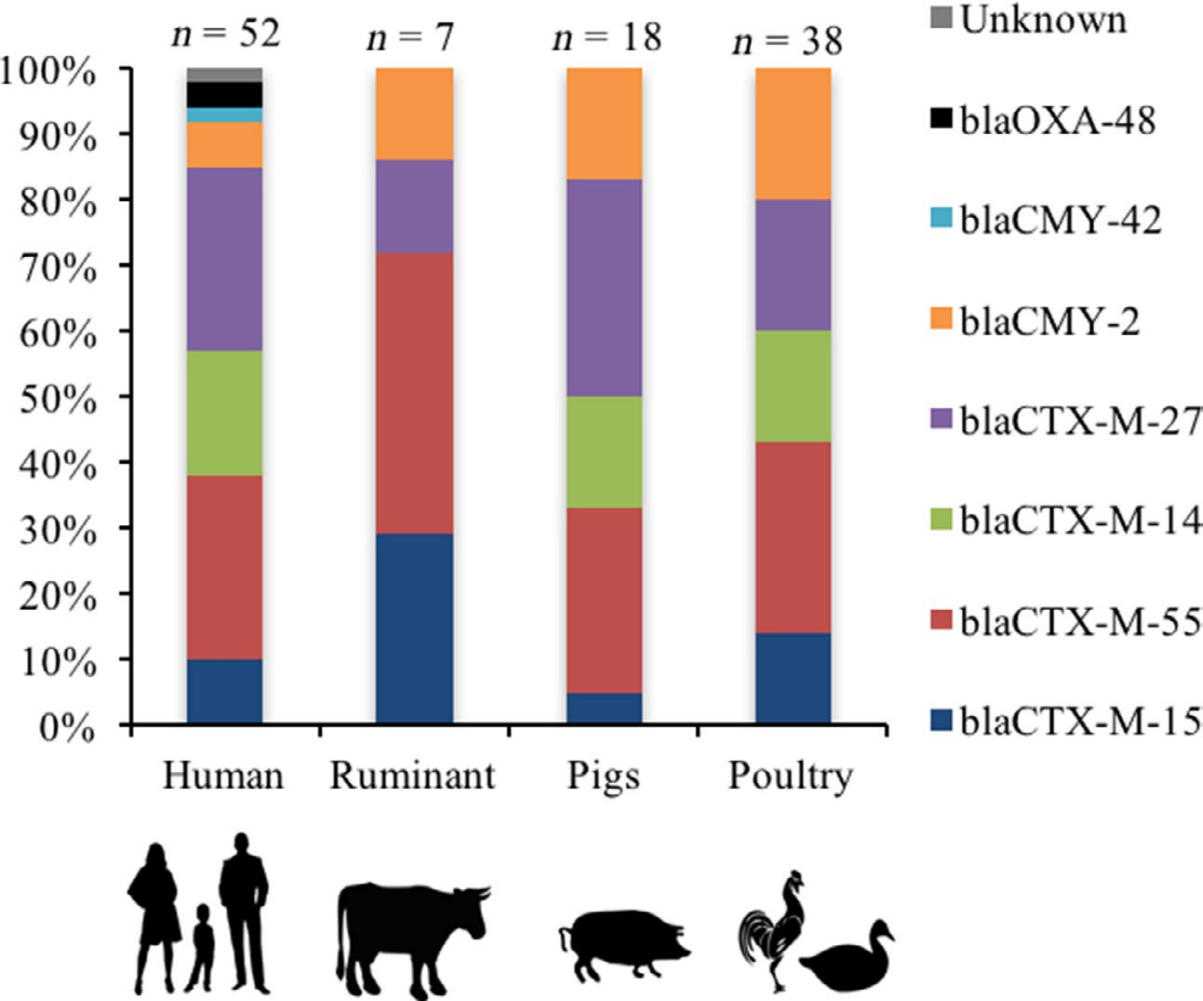
Distribution of carbapenemase‐ and extended‐spectrum cephalosporinase genes in *Escherichia coli *isolates (*n*) from humans and livestock living in 10 rural villages in Kampong Cham province, Cambodia in May 2011 [Colour figure can be viewed at http://www.wileyonlinelibrary.com]

All suspected carbapenem‐ and third‐generation cephalosporin‐resistant *E. coli* and *K. pneumoniae* isolated were analysed for the presence of carbapenemase, extended‐spectrum cephalosporinase and colistin resistance genes. Three CPE/K harboured the carbapenemase *bla*_OXA‐48_ gene. The two *bla*_OXA‐48_ *E. coli* isolates also harboured one additional AmpC/ESBL gene; *bla*_CMY‐2 _and *bla*_CTX‐M‐14_, respectively (Table 1). The one OXA‐48 *K. pneumoniae* isolate also harboured AmpC/beta‐lactamase genes *bla*_DHA‐1 _and *bla*_SHV‐11_ (Table 1). All 129 isolates that were resistant to third‐generation cephalosporins were verified to carry a ESBL or AmpC gene, with the exception of three *K. pneumoniae* which carried *bla*_SHV‐1 _and one *E. coli* in which no beta‐lactamase or ESBL gene was found, Table 1, Figure 1. All ESBL genes from 100 *E. coli* isolates were of CTX‐M‐type group 1 and 9, and especially *bla*_CTX‐M‐55 _(group 1) and *bla*_CTX‐M‐27_ (group 9) were frequently detected, with the others being *bla*_CTX‐M‐14 _(group 9) and *bla*_CTX‐M‐15 _(group 1). Seventeen *E. coli* isolates carried CMY‐2‐type genes; *bla*_CMY‐2 _and *bla*_CMY‐42_. In two *E. coli* isolates, both ESBL and AmpC genes were detected, Table 1.

In *K. pneumoniae*, 11 ESBL/AmpC genes were detected in nine isolates from human samples in the following distribution: *bla*_SHV‐2_ (4), *bla*_SHV‐11 _(1), *bla*_SHV‐28_ (1) *bla*_DHA‐1 _(2), *bla*_CTX‐M‐27_ (1), *bla*_CTX‐M‐14_ (1) and *bla*_CTX‐M‐15 _(1). From the chicken samples, four ESBL/AmpC genes were detected in four *K. pneumoniae* isolates *bla*_SHV‐12_, *bla*_CTX‐M‐14_, *bla*_SHV‐2_ and *bla*_DHA‐1 _(Table 1).

Two chickens and five humans were carrying both *E. coli* and *K. pneumoniae* that harboured ESBL/AmpC genes. The *E. coli* and *K. pneumoniae* isolates in the same individual harboured different ESBL/AmpC genes in all cases. One adult female carried three different isolates; one *E. coli* harbouring *bla*_OXA‐48 _and *bla*_CTX‐M‐14_; one *K. pneumoniae* harbouring *bla*_OXA‐48_, *bla*_SHV‐11_, *bla*_DHA‐1_ and one *K. pneumoniae* harbouring *bla*_CTX‐M‐14 _(Table 1). Two children and three chickens from the same household were sampled, and all were negative. Colistin resistance genes *mcr‐1‐like* or *mcr‐3‐like* were identified in 10 *E. coli* isolates and one *K. pneumoniae* isolate from two humans and nine livestock (Table 1).

### 3.2 Analyses of zoonotic risk factors associated with faecal carriage of ESCE/K in humans

To identify possible risk factors for faecal carriage of ESCE/K in humans, the head female in each of the 100 households was interviewed regarding household risk behaviour and meat consumption. Results from interviews were as follows; (a) livestock is slaughtered by someone in the household (76%), (b) livestock have access to sleeping and food preparation areas (57%), (c) unsafe water is consumed (36%), (d) hands are not washed with soap after handling animals (29%), (e) meat waste products are not burned or buried (21%), (f) animal manure is not collected daily indoors and outdoors (20%), (g) hands are not washed with soap before and after cooking (15%), and (8) undercooked meat is consumed (7%). The average number of days per month (d/m) that meat was consumed in households were: pork 5.7 d/m, beef 2.8 d/m, fish 22.5 d/m and poultry 2.2 d/m. Wildlife meat was consumed in 9/100 households, 1–10 d/m.

Based on the results of the univariable analysis, the following explanatory variables were selected for further analysis; livestock home slaughter, hand wash with soap after handling animals, burn or bury meat waste, daily collection of animal manure indoors and outdoors, consumption of undercooked meat and consumption of poultry. In the multivariable analysis, the household practice of not collecting animal manure indoors and outdoors daily was associated with increased odds of faecal carriage of ESCE/K isolates (*p* = 0.03, OR 2.19, 95% CI 1.07–4.47), whereas the household practice of not burning or burying meat waste was associated with decreased odds of faecal carriage of ESCE/K isolates (*p* = 0.01, OR 0.26, 95% CI 0.10–0.71).

### 3.3 Detection of CPE/K and ESCE/K

**Figure 2:**
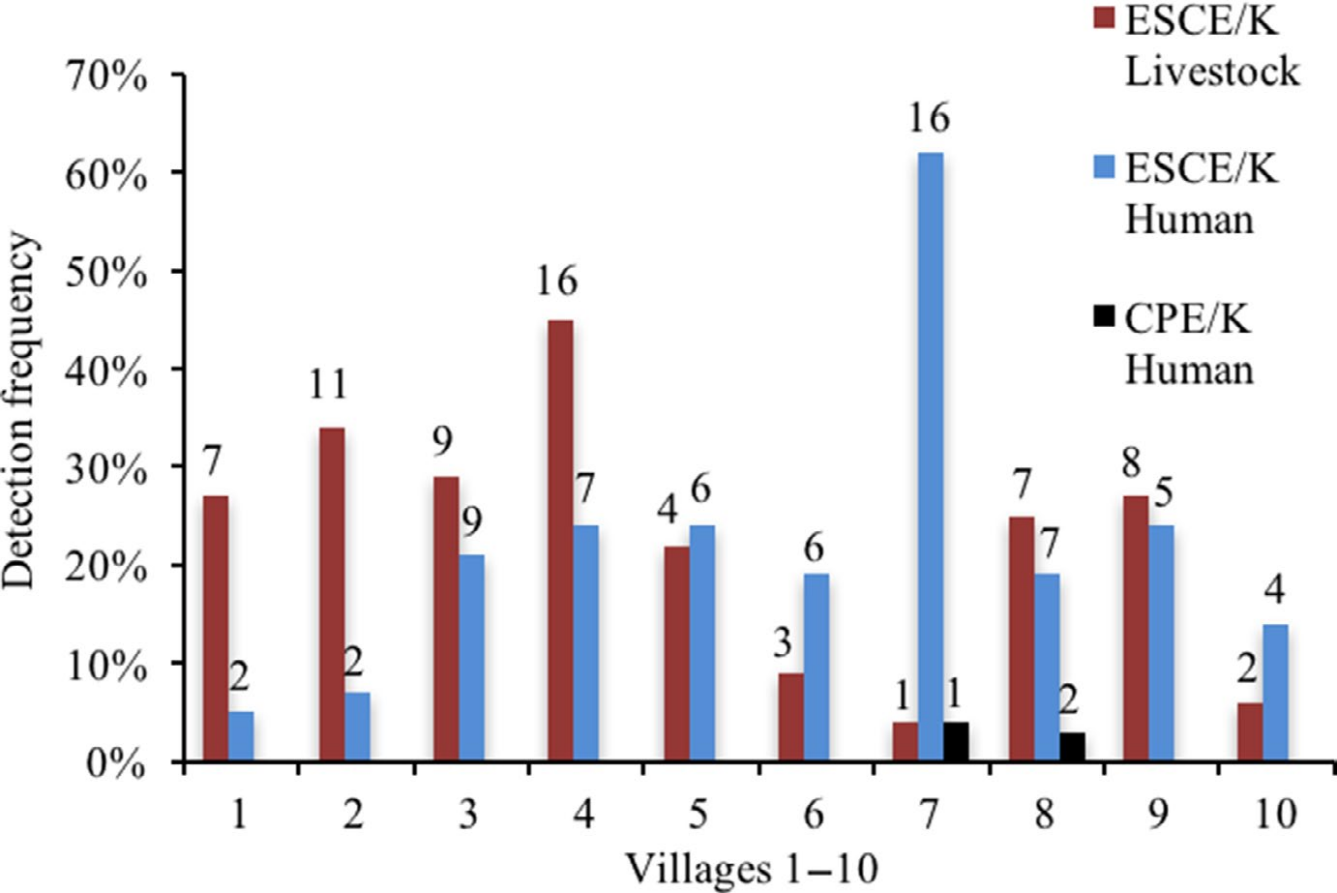
Detection frequency of carbapenemase‐ and extended‐spectrum cephalosporinase‐producing *Escherichia coli *and *Klebsiella pneumoniae *isolates in 10 rural villages in Kampong Cham province, Cambodia in 2011. Number of isolates indicated above each column [Colour figure can be viewed at http://www.wileyonlinelibrary.com]

**Figure 3:**
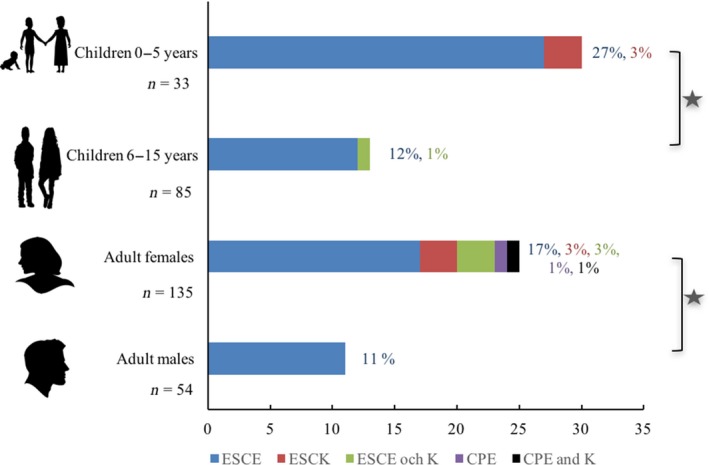
Detection frequencies of carbapenemase‐ and extended‐spectrum cephalosporinase‐producing *Escherichia coli *and *Klebsiella pneumoniae *in humans living in 10 rural villages in Kampong Cham province, Cambodia in May 2011, *n* = sampe size, * indicates statistical significant differences, *p* < 0.05 [Colour figure can be viewed at http://www.wileyonlinelibrary.com]

The overall detection frequency of CPE/K was 1% in humans and 0% in livestock. The overall detection frequency of ESCE/K isolates was 20% in humans and 23% in livestock, with the detection frequency ranging from 5% to 62% in humans and 4% to 45% in livestock in the 10 villages (Figure 2). The detection frequency of ESCE/K isolates in adult females (*n* = 135) and adult males (*n* = 54) was 23% and 11%, respectively, Figure 3. There was a significant (*p* = 0.03) difference between the combined detection frequencies of CPE/K and ESCE/K isolates in adult females compared with the combined detection frequencies of CPE/K and ESCE/K in adult males. In Figure 3, the detection frequencies in children are grouped based on age. No CPE/K isolates were detected in children, and the detection frequency of ESCE/K isolates was significantly (*p* = 0.04) higher, 30%, in age‐group 0–5 years (*n* = 33) than in age‐group 6–15 years (*n* = 85), 13%.

**Figure 4:**
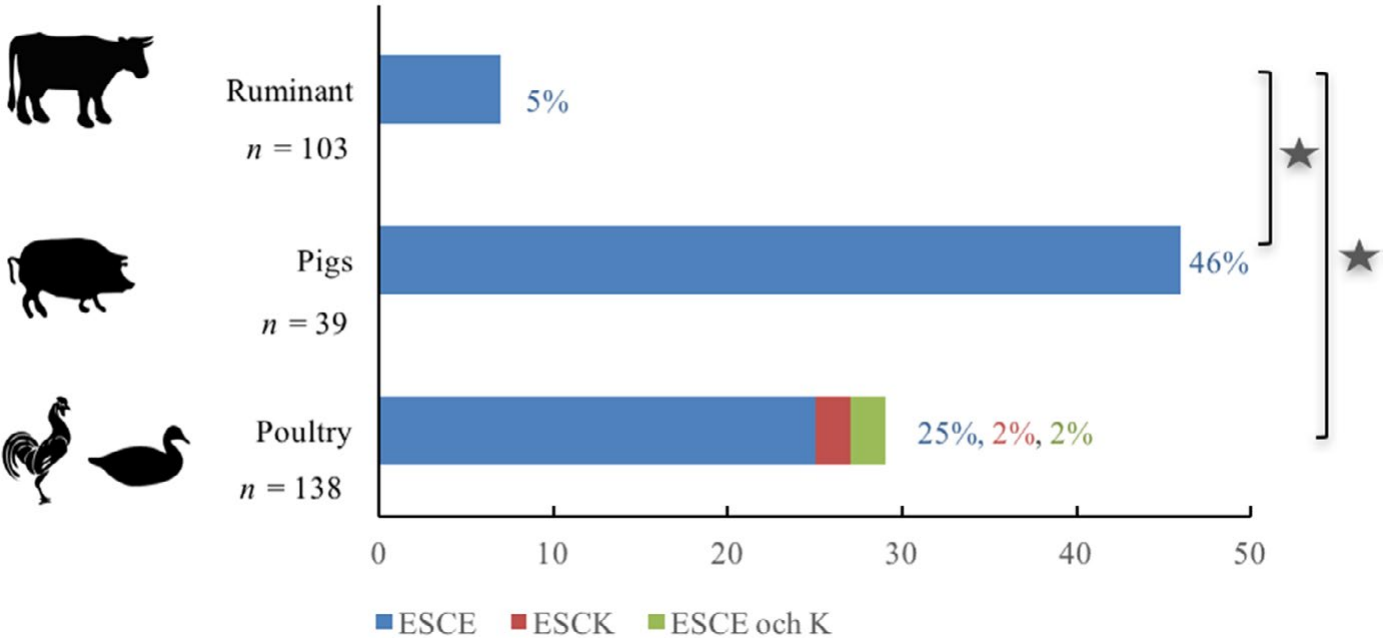
Detection frequencies of carbapenemase‐ and extended‐spectrum cephalosporinase‐producing *Escherichia coli *and *Klebsiella pneumoniae *in livestock living in 10 rural villages in Kampong Cham province, Cambodia in May 2011, *n* = sampe size, * indicates statistical significant difference, *p* < 0.02 [Colour figure can be viewed at http://www.wileyonlinelibrary.com]

In livestock (*n* = 285), no CPE/K isolates were detected, but 23% carried ESCE/K. In ruminant (*n* = 103) and pigs (*n* = 39), the detection frequencies of ESCE isolates were 7% and 46%, respectively, and no ESCK isolates were detected. In poultry (*n* = 138), the detection frequency of ESCE/K isolates was 28% (Figure 4)*.* The detection frequency was significantly higher in pigs and poultry compared with ruminants (both *p* < 0.0001), but the detection frequency was not significantly different between poultry and pigs (*p* = 0.10).

### 3.4 Susceptibility to other antibiotics

**Figure 5:**
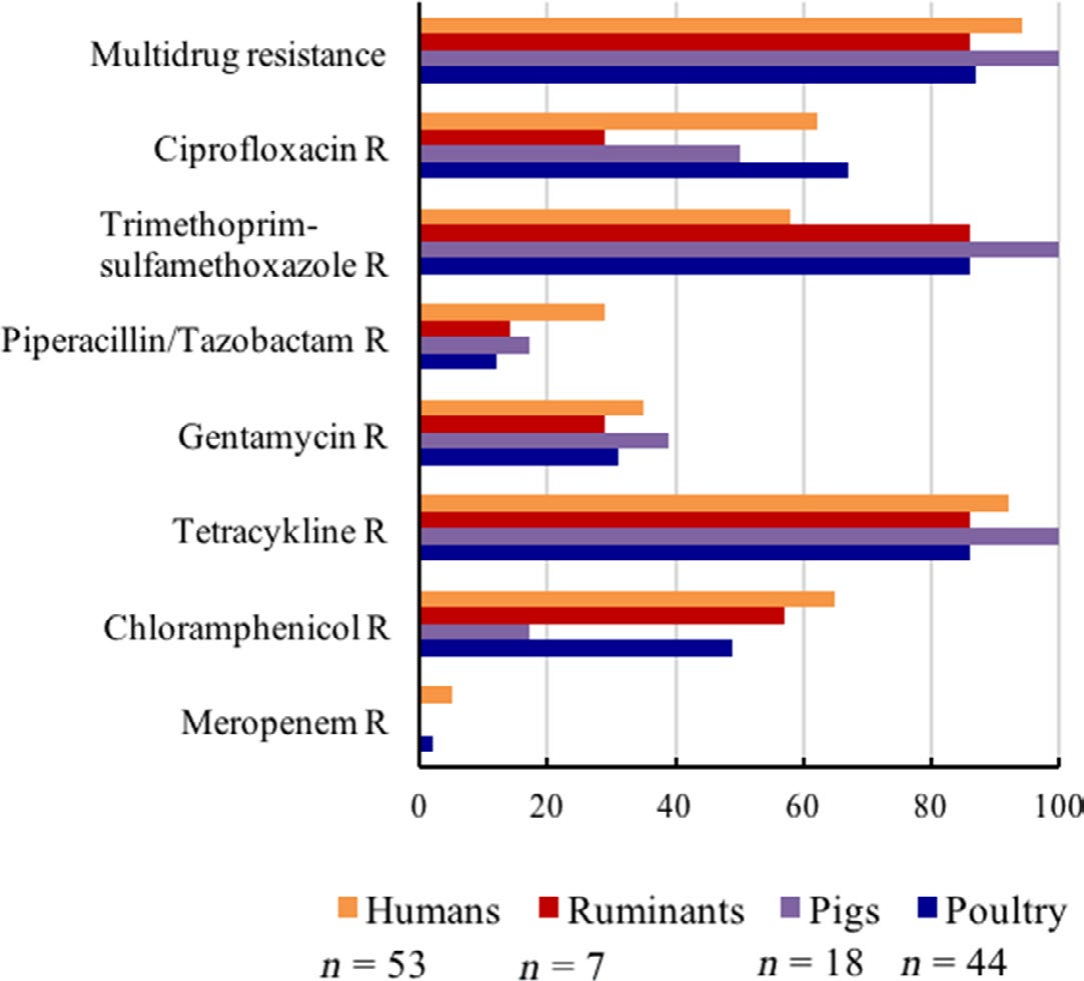
Antibiotic resistance in CPE/K and ESCE/K isolates (*n*) from humans and animals in rural Kampong Cham, Cambodia in 2011 determined by disc diffusion test and characterized as wild‐type or non‐wild‐type phenotype (R) [Colour figure can be viewed at http://www.wileyonlinelibrary.com]

Only CPE/K isolates and one ESCE expressed non‐wild‐type phenotypes to meropenem but resistance to other antibiotics was common (Figure 5 and Table 1). In total, 96% of the isolates were characterized as multidrug resistant, that is expressed a non‐wild‐type phenotype to ≥3 antibiotic classes. According to the clinical breakpoints provided by CLSI, 92% of the isolates were characterized as multidrug resistant.

## 4 DISCUSSION

To the best of our knowledge, this is the first published report of *bla*_OXA‐48 _in *E. coli* and *K. pneumoniae* in Cambodia. The presence of *bla*_OXA‐48 _harbouring *E. coli* and *K. pneumoniae* in the community is of special concern because carbapenems are the last line of defence against invasive multiresistant Gram‐negative bacteria (Papp‐Wallace, Endimiani, Taracila, & Bonomo, 2011). However, the detection frequency of carbapenem‐resistant *E. coli/K. pneumoniae* in rural Cambodia in 2011 was still low, 1% in humans and not detected in livestock. Community carriage of *bla*_OXA‐48 _harbouring Enterobacteriaceae is rare, but has been reported from humans in Lebanon (Beyrouthy et al., 2014) and Switzerland (Zurfluh et al., 2015). *Bla*_OXA‐48 _harbouring isolates expressed a non‐wild‐type phenotype to meropenem, ciprofloxacin, sulfamethoxazole/trimethoprim, piperacillin/tazobactam, tetracycline and chloramphenicol but colistin resistance genes were not detected.

In this study, the combined detection frequency of CPE/K and ESCE/K in stool from adult females was significantly higher compared with adult males. This contrasts to community carriage in Western Europe where no difference between genders was observed (Ny et al., 2017; Valenza et al., 2014; Wielders et al., 2017). Furthermore, ESBL colonization in male neonatal children was more common compared with female neonatal children in an Israeli hospital (Leikin‐Zach et al., 2018). There are no obvious biological reasons for the observed difference in community carriage between sexes, and the explanation could be local gender‐related behaviour leading to transmission between populations. In the current study population, women are often more responsible for the care of poultry and pigs (high level ESCE/K colonized livestock), while men generally take care of more valuable livestock such as ruminants (low level ESCE/K colonized livestock) (Osbjer et al., 2015). It has been previously shown that close contact with poultry increased community carriage of ESCE in Dutch humans (Huijbers et al., 2014). Furthermore, women are generally responsible for the care of young children and the current study identify that young children aged 0–5 years were more prone to carry ESCE/K (30%) compared with older children ages 6–15 years (13%). Young children are incontinent, have less developed hygiene and intimate contact with the environment, animals and their caregiver. Thus, it is reasonable to assume that transmission of ESCE/K occurs between adult females and children and/or poultry/pigs and children. A Swedish study found that children below 3 years, in contrast to older children and adults, were colonized with the same ESCE as the family dog (Ljungquist et al., 2016). In addition, it has been found that there is a six‐fold increased risk of ESBL‐producing Enterobacteriaceae colonization in underweight newborns if the mother is a carrier (Denkel et al., 2014). Little is known about ESCE/K in different age groups of children though similar to our results, stool samples from 519 children in the US revealed a higher colonization frequency of ESCE/K amongst children <5 years (5.7%) compared with children >5 years (1.7%) (Islam et al., 2017). The overall detection frequency amongst children (age 0–15) in rural villages in Kampong Cham province, Cambodia, in 2011 was 19%. Other reports examining community carriage in healthy children have found varying prevalence frequencies; 2.9% in Sweden 2010 (Kaarme, Molin, Olsen, & Melhus, 2013), 20% in Sweden 2016 (Kaarme et al., 2018), 2.7% in Portugal 2008 (Guimaraes et al., 2009) and 49.6% in infants in Lebanon 2013 (Hijazi, Fawzi, Ali, & Abd El Galil, 2016a). The observed difference between children age 0–5 years (30%) and 6–15 years (13%) was statistically significant using the chi‐square test, but due to a small sample size in the 0–5 years group (*n* = 33), the 95% confidence intervals are overlapping between the two groups, and our results should be interpreted with caution.

Our results show that certain household practices in rural Cambodia are potential risk factors for faecal carriage of ESCE/K*.* The practice of daily collection of animal manure indoor and outdoor decreased the risk of faecal carriage of ESCE/K, indicating that removing animal manure reduces the environmental exposure to antibiotic‐resistant bacteria. The practice of either burning or burying meat waste in a household was a risk factor for faecal carriage of ESCE/K. This is perhaps contradictory as meat waste could be contaminated with antibiotic‐resistant bacteria (Lazarus, Paterson, Mollinger, & Rogers, 2015), and burning or burying meat waste would limit the exposure. However, burning of burying meat waste could be a confounding factor to for example home slaughter or other actions increasing contact with raw meat, but home slaughter was not significantly associated with faecal carriage in this study. The possible impact of home slaughter is supported by a study in Cameroon showing that ESBL‐producing *K. pneumoniae* isolates disseminate from animals to abattoir workers (Founou et al., 2018). There were no significant associations between meat consumption and faecal carriage of ESCE/K, which contrast previous studies that have shown regular consumption of meat and consumption of undercooked meat to be risk factors for community carriage of ESBL‐producing Enterobacteriaceae (Hijazi et al., 2016b; Niumsup et al., 2018).

The same ESBL/AmpC genes detected in *E. coli/K. pneumoniae* from human and livestock faeces in our study (*bla*_CTX‐M‐55_, *bla*_CTX‐M‐27_, *bla*_CTX‐M‐15_, *bla*_CTX‐M‐15_, *bla*_CMY‐2 _and *bla*_DHA‐1_) were detected in *E. coli/K. pneumoniae* from bloodstream infections in Phnom Penh, Cambodia, between 2007 and 2010 (Vlieghe et al., 2015). This indicates that the gut serves as a reservoir for extra‐intestinal pathogenic *E. coli*, which has been previously suggested (Carlet, 2012). It is important to consider that the gene variation in the current study might be underestimated as only one colony was selected on each agar plate. In one *E. coli* and two *K. pneumoniae*, no extended‐spectrum beta‐lactamase gene could be identified and further molecular analysis is required to establish whether a less common or a novel ESBL gene can explain the phenotype.

The zoonotic potential of ESCE/K and CPE/K is of concern, and previous work has shown that the awareness of zoonotic risks of antibiotic‐resistant bacteria is low in the current study population (Osbjer et al., 2015). Transmission of bacteria and/or mobile genetic elements between populations through contact and environmental exposure seem likely, as the same ESBL/pAmpC genes were detected in humans and livestock and the antibiotic resistance profile was similar in ESCE/K isolates. However, additional molecular work is needed to better understand relatedness between isolates in different hosts, but was not within the scope of this study.

The demonstrated community carriage of ESCE/K in humans and livestock in Cambodia (20% in humans and 23% in livestock) is similar to a recent report on ESBL‐producing *E. coli* colonization in chicken farmers (20%) and chickens (35%) from Vietnam (Nguyen et al., 2019) but lower than in reports from Thailand (62%) and Vietnam/Laos (41%–70%) (Nakayama et al., 2015; Niumsup et al., 2018). The lower detection frequency in our study could be due to the rural habitat of the sampled population and that previous thawing of some samples has led to an underestimation of human carriage. The difference in ESCE/K detection between villages could partly be explained by skewed sampling. Poultry and pigs were sampled in high numbers in the high detection village number 4, as opposed to the low detection village number 7, which contained many samples from ruminants. In contrast, the detection frequency in humans was very high in village 7. Owning ruminants is associated with high wealth in Cambodia (Osbjer et al., 2015), and wealthy families might be more likely to travel and use medicine like antibiotics, both risk factors for acquiring antibiotic‐resistant bacteria (Karanika et al., 2016). The high detection frequencies of ESCE/K in poultry and pigs could be related to transmission and inappropriate use of antibiotics. Pigs were often kept in crowded confinement (Osbjer et al., 2015), which allows for frequent transmission of bacteria. Previous studies have found that antibiotic use in the pig and poultry industry in Cambodia is widespread and uncontrolled (Om & McLaws, 2016; Ström, Boqvist, et al., 2018). ESCE is also frequent in poultry production in Europe, even in countries with low antibiotic resistance burden, and the prevalence is mainly related to vertical transmission (Blaak et al., 2015; Borjesson et al., 2016). The ESCE/K in pig manure is an environmental hazard, as pig farmers in Cambodia often dump the pig manure in the environment (Ström, Albihn, et al., 2018).

Colistin resistance genes were identified in poultry, pigs and humans. The *mcr*‐1 gene has been previously detected in stool sample from a Cambodian child (Stoesser, Mathers, Moore, Day, & Crook, 2016) but to our knowledge this is the first finding of *mcr‐3‐like* gene in the country. Resistance to colistin is particularly worrisome as colistin can be the last available treatment for CPE/K (Falagas, Karageorgopoulos, & Nordmann, 2011). A study on backyard chicken farms in Vietnam concluded that detection of *mcr‐1*‐carrying bacteria in chicken samples was associated with colistin use and that detection in human samples was associated with exposure to *mcr‐1*‐positive chickens (Trung et al., 2017). Through interviewing pig farmers in Cambodia, Ström, Boqvist, et al. (2018)) found that antibiotic use, including colistin, was common in pig farms and sometimes used as prophylactic treatment.

## 5 CONCLUSIONS

Carbapenemase and colistin resistance genes were present in the Cambodian community to a low extent in 2011, but continuous surveillance is necessary as dissemination of multidrug‐resistant bacteria is a dynamic process. Faecal carriage of *E. coli* and *K. pneumoniae* harbouring extended‐spectrum cephalosporinase genes were common in rural Cambodia, with more frequent occurrence in women and young children. Environmental exposure and contact with animal manure and slaughter products were risk factors for intestinal colonization of ESCE/K, suggesting that farming households and animal health workers should be further educated on hygiene precautions to limit such exposure.

## CONFLICT OF INTEREST

None to declare.
